# Potential immunosuppressive clonal hematopoietic mutations in tumor infiltrating immune cells in breast invasive carcinoma

**DOI:** 10.1038/s41598-023-40256-9

**Published:** 2023-08-12

**Authors:** Ramu Anandakrishnan, Ian J. Zyvoloski, Lucas R. Zyvoloski, Nana K. Opoku, Andrew Dai, Veneeth Antony

**Affiliations:** 1 Edward Via College of Osteopathic Medicine, Biomedical Sciences, Blacksburg, VA USA; 2 Virginia Tech, Blacksburg, VA USA; 3 Gibbs Cancer Center and Research Institute, Spartanburg, SC USA; 4 Severna Park HS/MS, Severna Park, MD USA

**Keywords:** Breast cancer, Cancer genomics, Tumour immunology, Genome informatics, Cancer genomics, Genetic markers, Genome, Genomic instability, Genomics, Immunogenetics, Immune evasion, Immunogenetics, Diagnostic markers

## Abstract

A hallmark of cancer is a tumor cell’s ability to evade immune destruction. Somatic mutations in *tumor cells* that prevent immune destruction have been extensively studied. However, somatic mutations in *tumor infiltrating immune (TII) cells*, to our knowledge, have not been previously studied. Understandably so since normal hematopoiesis prevents the accumulation of somatic mutations in immune cells. However, clonal hematopoiesis does result in the accumulation of somatic mutations in immune cells. These mutations cannot “drive” tumor growth, however, they may “facilitate” it by inhibiting an effective anti-tumor immune response. To identify potential immunosuppressive clonal hematopoietic (CH) mutations in TII cells, we analyzed exome and RNA sequencing data from matched tumor and normal blood samples, and single-cell RNA sequencing data, from breast cancer patients. We selected mutations that were somatic, present in TII cells, clonally expanded, potentially pathogenic, expressed in TII cells, unlikely to be a passenger mutation, and in immune response associated genes. We identified eight potential immunosuppressive CH mutations in TII cells. This work is a first step towards determining if immunosuppressive CH mutations in TII cells can affect the progression of solid tumors. Subsequent experimental confirmation could represent a new paradigm in the etiology of cancer.

## Introduction

Although considerable progress has been made in reducing mortality rate in cancer patients, it remains a leading cause of death, second only to heart disease^1^. Clearly, there is an urgent need for more effective treatments. Cancer is a complex disease caused by a combination of several different factors^2–4^. One of the hallmarks of cancer is a tumor cell’s ability to avoid immune destruction^2^. The mechanisms by which tumor cells avoid immune destruction have been extensively studied^5–7^. These mechanisms include mutations in tumor cells that allow them to evade detection by immune cells, inhibit immune cell recruitment/infiltrations, induce immune cell apoptosis, and produce factors that inhibit immune response. However, the potential effect of somatic mutations in tumor infiltrating immune (TII) cells, to our knowledge, have not been previously studied. This is understandable since somatic mutations do not generally accumulate during normal hematopoiesis where immune cells are constantly replaced by fresh cells from hematopoietic stem cells^8^. However, in a relatively recently discovered phenomenon, clonal hematopoiesis, mutations in specific genes can lead to the clonal proliferation of individual cells resulting in a significant clonal population with somatic mutations^9,10^.

**Figure 1:**
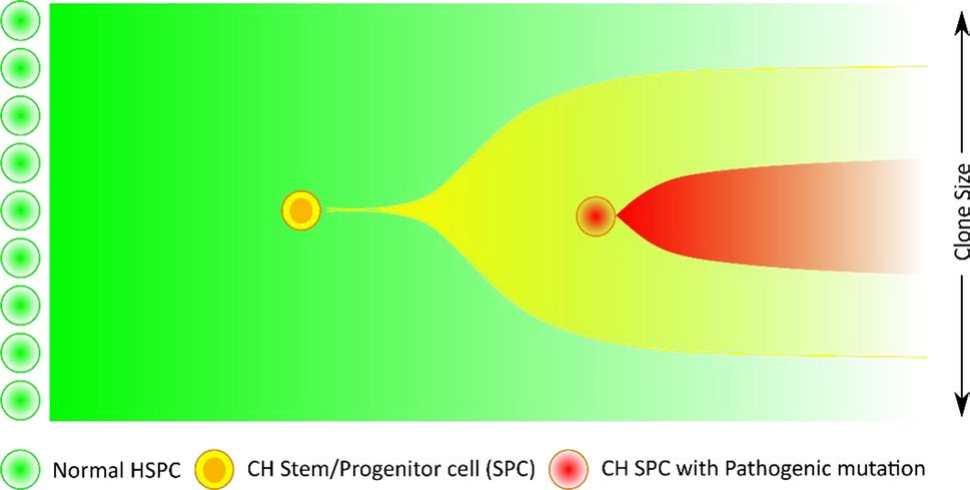
Clonal expansion of pathogenic mutations in TII cells. Increased cell survival and/or growth due to clonal hematopoietic (CH) mutations in hematopoietic stem/progenitor cells (HSPC) result in the expansion of a clonal population. Secondary pathogenic mutations within this population can result in the presence of the mutation in a large enough proportion of immune cells for pathogenesis.

Mutations in genes, such as DNA Methyltransferase 3 Alpha (*DNMT3A*), Ten-Eleven Translocation 2 (*TET2*), and Additional Sex Combs-Like Protein 1 (*ASXL1*), have been shown to cause clonal hematopoiesis, which is characterized by these somatic mutations being present in greater than 2% of peripheral blood cells^11^. Three possible mechanisms have been proposed for clonal hematopoiesis. The mutation in a hematopoietic stem or progenitor cell causes (1) increased self-renewal; (2) increased number of self-renewal cycles required to become a committed progenitor, or (3) increased epigenetic or transcriptional heterogeneity leading to clonal selection of highly proliferative states^10,12,13^. Clonal hematopoiesis has been implicated in hematologic^14^ and cardiovascular^15^ malignancies. Over 10% of adults over age 70 have clonal hematopoiesis^16^. In addition, mutations associated with clonal hematopoiesis frequently occur in TII cells (14–65% of study samples)^17–21^. A recent study has identified 70 genes that may “drive” clonal expansion^22^. However, clonal hematopoiesis does not by itself result in these malignancies. Secondary mutations are required to produce a disease state^9^. A longitudinal study of a patient who died of secondary acute myeloid leukemia (sAML) exemplifies clonal hematopoietic (CH) progression^14^. Clonal hematopoiesis due to a *DNMT3A* mutation was detected in blood samples taken at age 64. At age 69 the patient was diagnosed with myelodysplastic syndrome (MDS) due to a secondary *RUNX1* (Runt-Related Transcription Factor 1) mutation in a CH clone. At age 72 the patient was diagnosed with sAML, the cause of death, due to a secondary *FLT3* (Fms-Like Tyrosine Kinase 3) mutation in a *DNMT3A-RUNX1* mutant subclone. The general mechanism by which a secondary pathogenic mutation can accumulate in a clonally expanding immune cell population is illustrated in Fig. 1.

As with clonal hematopoiesis related myeloid malignancies, secondary mutations in clonal hematopoietic cells (CH mutations) could also result in the accumulation of cells with immunosuppressive mutations (Fig. 1), thus inhibiting an effective anti-tumor immune response. As an intuitive rationale consider the following. Immunodeficiency, either due to germline defects or immunosuppressive treatments, is well known to increase cancer incidence^23,24^. In addition, altering the expression of specific immune cell genes can affect their anti-tumor immune function^25,26^. Similarly, secondary immunosuppressive mutations in a significant number of TII cells due to clonal hematopoiesis would represent some degree of immunodeficiency, which could affect the progression of solid cancers. 

As a first step towards investigating the above hypothesis, in this study we identified potential immunosuppressive mutations in TII cells. To identify these mutations, we downloaded and analyzed exome and RNA sequencing data for 1,064 breast invasive carcinoma (BRCA) samples from the cancer genome atlas (TCGA) database^27^ and single cell RNA sequencing data for 26 breast cancer tumor samples from the gene exchange omnibus (GEO) database^28^. Mutations were selected based on the following seven criteria. The mutations must be (1) somatic, (2) in TII cells, (3) clonally expanded in blood (CH), (4) potentially pathogenic, (5) in a gene expressed in immune cells, (6) unlikely to be a passenger mutation, and (7) potentially immunosuppressive. 

## Results

**Figure 2:**
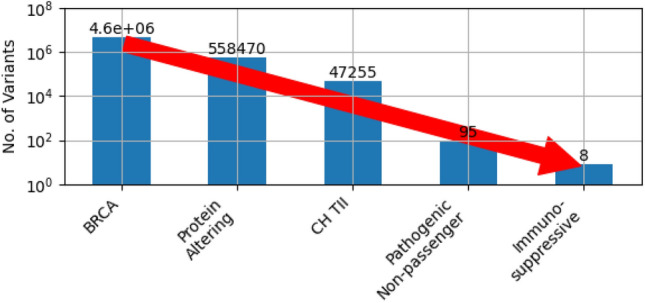
Four stage approach for identifying potential immunosuppressive CH mutations in TII cells. (1) Select protein altering mutations which are more likely to be pathogenic than intergenic, intronic and synonymous mutations. (2) Select CH mutations in TII based on VAF in matched blood and tumor samples. (3) Select potential pathogenic (non-passenger) variants (rare variants occurring frequently in BRCA, predicted to be deleterious, and expressed in TII cells). (4) Select variants in genes experimentally shown to affect immune function. With these criteria, we identified eight potential immuno-suppressive CH mutations in TII cells, out of 4.6 million different variants in 1,064 BRCA samples.

Our approach for identifying potential immunosuppressive CH mutations in TII consisted of four stages as shown in Fig. 2. (1) We selected protein altering mutations, which are more likely to be pathogenic than non-coding and synonymous mutations. (2) Clonally expanded somatic mutations in TII were identified based on variant allele fraction (VAF). (3) Potential pathogenic non-passenger mutations were identified based on frequency of occurrence in the genome aggregation database (genomAD). In addition, we confirmed that the gene is expressed in immune cells in the breast cancer tumor microenvironments using single-cell RNA sequencing (scRNA-seq) data. We also excluded potential passenger mutations based on several criteria, such as mutations in genes that are naturally hypermutated during immune response. (4) Finally, we selected potential immunosuppressive mutations based on pathways affected by significantly differentially expressed genes and published experimental evidence supporting their role in immune response. Overall, our selection criteria were designed to reduce the possibility of including passenger mutations even at the risk of excluding additional potential immunosuppressive CH mutations in TII cells. Based on the above criteria, we identified eight potential immunosuppressive CH mutations in TII cells (Table 1).

**Table 1:** Potential immunosuppressive CH mutations in TII cells. Some of the key selection criteria used to select each variant are listed here. Variant allele fraction (VAF) shows mean and standard deviation for the samples in which the variant is detected. N = 1,064 samples for sample fraction. N > 100,000 sequences for gnomAD frequency. For % of TII cells in which the gene was expressed, the TII and total cell counts from single-cell RNA sequencing of the tumor microenvironment for 26 breast cancer patients, are shown in parentheses.

Gene	Variant	Tumor VAF	Blood VAF	Sample Fraction	gnomAD Freq	% of TII cells in which gene was expressed
*C1GALT1C1*	p.V276G	0.18 ± 0.03	0.14 ± 0.05	0.0705	2E − 5	23% (2,810/12,075)
*DPP4*	p.V354G	0.18 ± 0.03	0.13 ± 0.06	0.1156	4E − 6	46% (1,110/2,390)
*EIF4EBP1*	p.D55H	0.07 ± 0.04	0.05 ± 0.02	0.0855	0	28% (7,596/27,530)
*EIF4EBP1*	p.R56W	0.07 ± 0.03	0.05 ± 0.02	0.0902	8E − 6	28% (7,596/27,530)
*EIF4EBP1*	p.R63W	0.07 ± 0.04	0.05 ± 0.02	0.0770	4E − 6	28% (7,596/27,530)
*EIF4EBP1*	p.S65L	0.08 ± 0.04	0.05 ± 0.02	0.0761	4E − 6	28% (7,596/27,530)
*KIF15*	p.T334P	0.17 ± 0.04	0.13 ± 0.05	0.0695	9E − 6	38% (603/1,572)
*UBE2N*	p.P63A	0.07 ± 0.03	0.08 ± 0.04	0.1137	0	41% (15,806/38,958)

### Protein altering variants

Matched blood and tumor samples from 1,064 breast invasive carcinoma (BRCA) patients were used for this study. BRCA cases consisted of 63.6% HR + /HER − , 21.6% HR − /HER − , 11.44% HR + /HER2 + , and 3.4% HR − /HER2 + (SI Figure S1A), which are significantly different from the 87.4%, 13.2%, 12.6%, and 5.1%, respectively in the surveillance, epidemiology, and end results (SEER) report for the US^29^. The median age at diagnosis of 58 for BRCA cases (SI Figure S1B), is lower than the median age of 63 in the SEER report. BRCA cases consisted of 73.0% Stage I and II cases and 24.5% Stage III and IV cases (SI Figure S1C) compared to 65.1% Localized and 33.0% Regional/Distant cases in the SEER report. These differences will need to be accounted for in any future studies considering the mutations identified here as potential risk markers.

A total of 4,579,609 different mutations were detected in BRCA exome sequencing data. The vast majority of these, such as synonymous, intergenic and intron mutations, are less likely to be pathogenic. Therefore, we excluded these mutations, limiting subsequent analysis to protein altering mutations, which are more likely to be pathogenic. In addition, for this analysis we did not consider epigenetic modifications and copy number variations since their pathogenicity is in general unclear. Protein altering mutations included nonsense, missense, insertion, deletion, and splice site variants. Of the 4,579,609 different mutations, 558,470 (12%) were in coding regions. Of the coding region variants 445,132 (80%) were protein altering mutations (SI Figure S1D).

### Clonally expanded somatic mutations in tumor infiltrating immune cells

**Figure 3:**
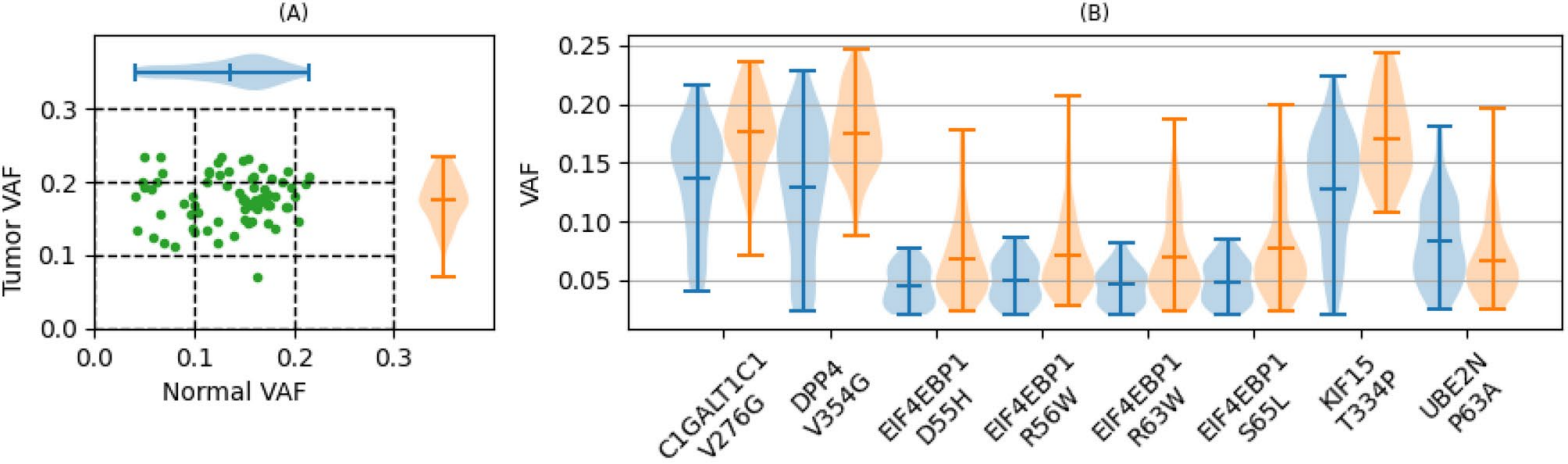
CH variants in TII cells. (**A**) Variant allele fraction (VAF) in matched blood and tumor samples for *C1GALT1C1* p.V276G. (**B**) Distribution of VAF for each of the eight potential immunosuppressive CH variants in TII. Center markers are the mean and end markers are the minimum and maximum. VAF distributions for normal blood samples are shown in blue and for tumor samples in red. VAF 95% confidence intervals for each sample is shown in SI Table S2.

Clonally expanded somatic mutations were identified by the variant allele fraction (VAF) in matched tumor and blood sample. A lower threshold of VAF > 2% is generally accepted as indicative of clonal hematopoiesis ^16^. With this threshold the estimated false positive rate due to sequencing errors is < 1%^30,31^. This threshold also ensures that the identified mutations are not from circulating tumor DNA or cell free DNA, which can comprise up to 1% of blood sample DNA^32,33^. An upper threshold of VAF < 25% was used to select for somatic variants and exclude germline variants. Mutations that occur with 2% < VAF < 25% *in both blood and tumor samples* are indicative of CH mutations in TII cells. It is highly improbable that the same variant would arise independently in blood and tissue cells, therefore it is reasonable to assume that the mutation occurred in blood cells and was detected in tumor samples due to the presence of infiltrating immune cells in the tumor microenvironment. For example, the CH VAF in blood and tumor samples for the Core 1 Beta3-Galactosyltransferase-Specific Molecular Chaperone (*C1GALT1C1*) p.V276G variant, one of the eight potential immunosuppressive mutations in TII cells (Table 1), varies from 7.1–23.5% for tumor and 4.1–21.6% for blood samples (Fig. 3A). The VAF for all 8 mutations are summarized in Fig. 3B and in Table 1. Of the 558,470 protein altering mutations, 47,255 (8.5%) were clonally expanded somatic mutations in TII cells based on the above criteria (Fig. 2).

### Likely pathogenic non-passenger mutations

To maximize the likelihood that the mutations identified are pathogenic and not passenger mutations, we selected mutations that satisfied the following additional criteria: rare mutations that frequently occurred in BRCA samples, mutations in genes that are expressed in tumor infiltrating immune cells based on scRNA-seq data, and deleterious or damaging mutations. Again, we emphasize that although the goal is to filter out passenger mutations, the mutations identified are not “driver” mutations. Since the mutations are in immune cells and not in tumor tissue cells, they will not directly drive tumor growth. Instead, the mutations identified here could “facilitate” tumor growth by inhibiting an effective anti-tumor immune response.

**Figure 4:**
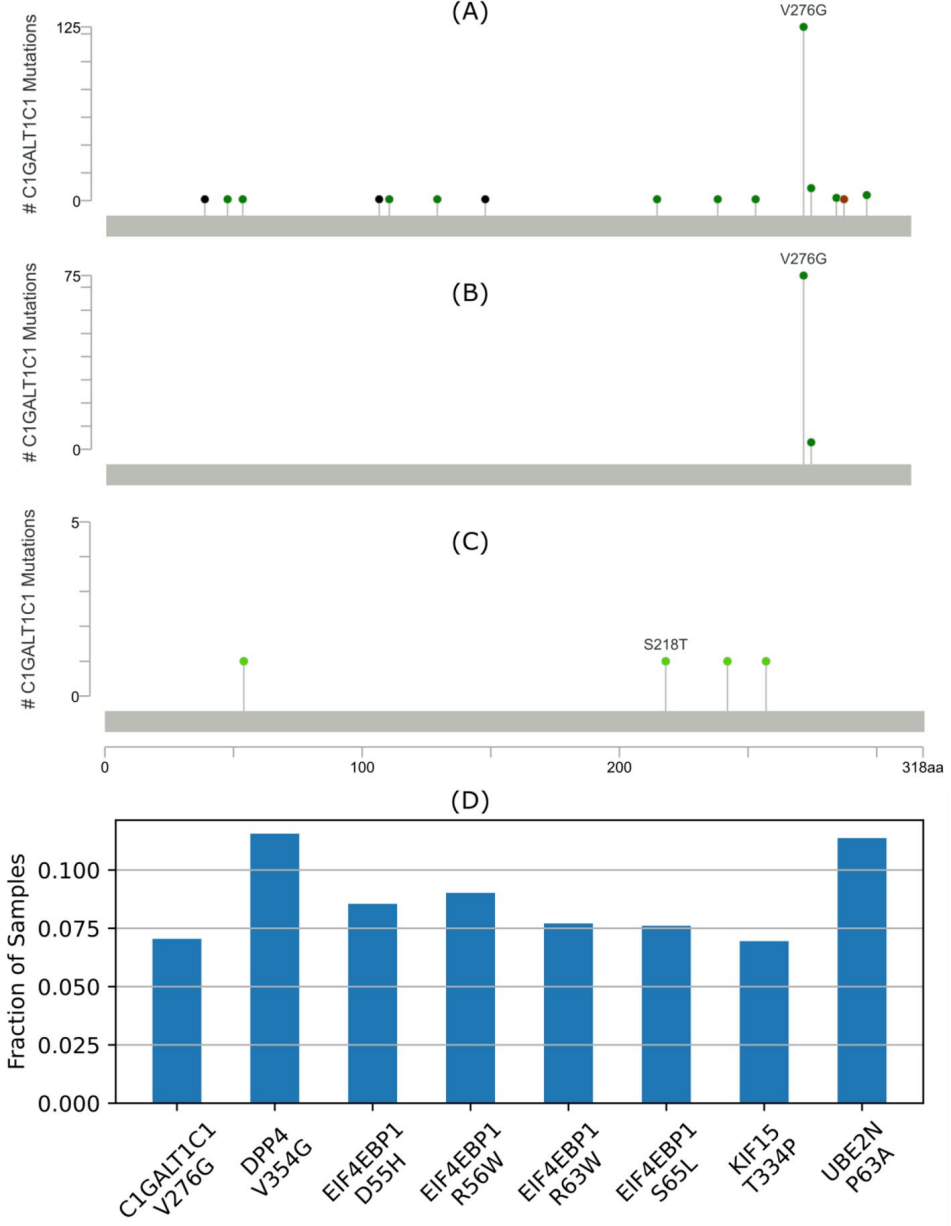
Fraction of samples with variants. N = 1,064 samples. (**A**) All variants in *C1GALT1C1*. (**B**) *C1GALT1C1* CH variants in TII cells. (**C**) Open access *C1GALT1C1* variants (tumor sample variants not detected in matched blood samples) reported by cBioPortal. (**A**–**C**) Missense, frame-shift and in-frame mutations are shown by green, black and brown dots, respectively. (**D**) Fraction of samples with each of the eight immunosuppressive CH variants in TII cells listed in Table 1.

The genome aggregation database (gnomAD) includes variant information from over 100,000 exome sequences. Rare variants in gnomAD (allele population frequency < 0.01%) suggests that the variant may have a deleterious effect on gene function. In tumor cells, rare mutations can be a result of rapidly dividing tumor cells with defective DNA repair mechanisms. Many of these mutations may be passenger mutations, with no effect on tumor progression. However, non-tumor immune cells generally do not contain defective DNA repair mechanisms and are not rapidly dividing. Therefore, the relatively frequent occurrence (> 5%) of such rare mutations in BRCA blood samples, suggests that the mutation could affect cell function^34^. For example, the p.V276G variant in *C1GALT1C1* occurs in 75 (7%) of 1,064 matched BRCA normal blood samples (Fig. 4A), compared to 2 (0.0019%) of 105,263 sequences in gnomAD. The frequency of the eight potential immunosuppressive CH mutations in TII cells range from 7.05 – 11.56% in BRCA normal blood samples (Fig. 4D), compared to the 0 – 0.002% in gnomAD sequences (Table 1). As noted in the Methods section, we used protected TCGA data which includes mutations in blood samples (Fig. 4A) that would be excluded in open access databases since they are considered potential germline mutations that may identify the sample donor (Fig. 4C). For this analysis we selected CH mutation in TII cells (Fig. 4B). Figure 4A mutations not included in Fig. 4B,C are non-CH variants in TII (VAF ≤ 2%) or potential germline mutations (VAF ≥ 25%).

**Figure 5:**
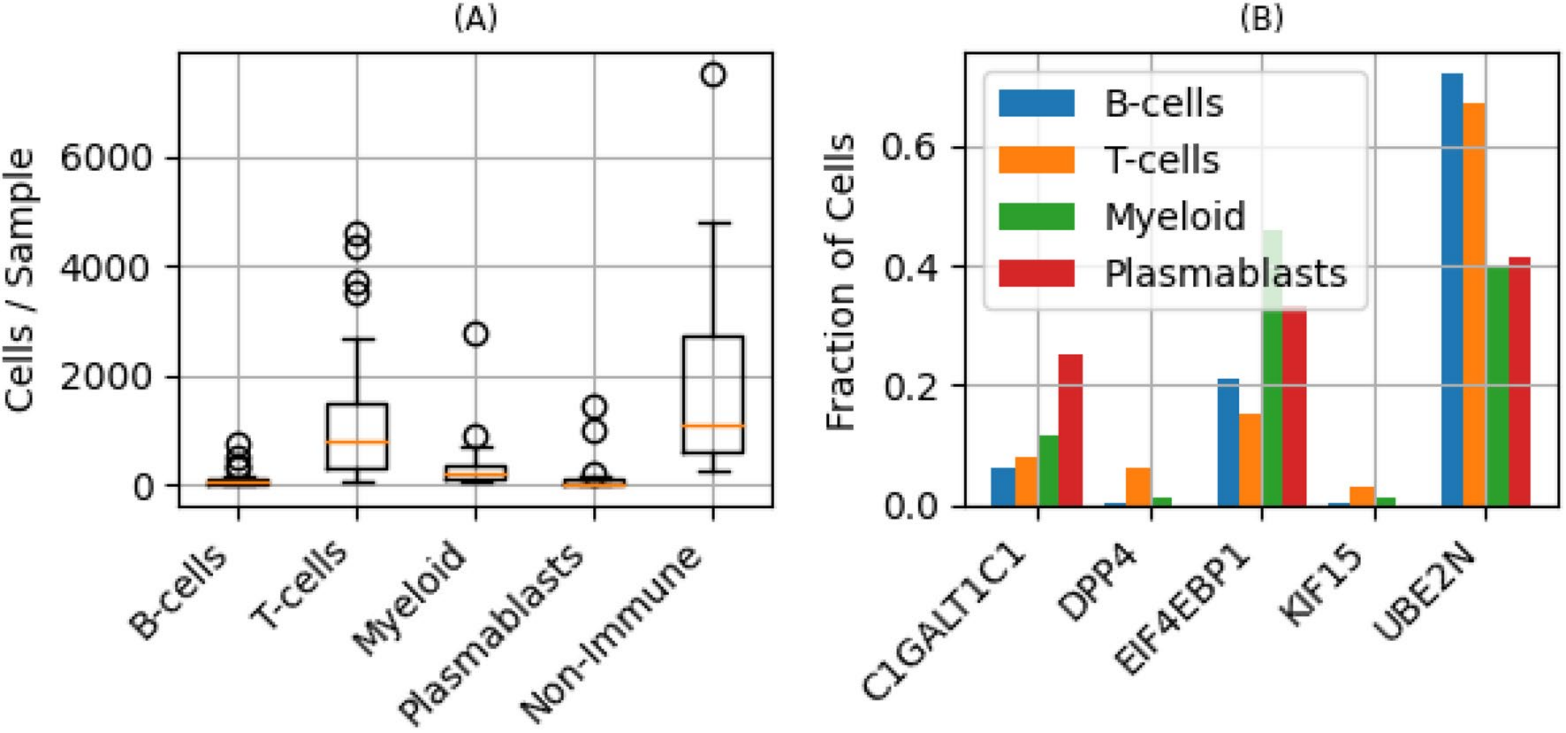
Genes associated with potential immunosuppressive CH mutations in TII cells expressed in immune cells. (**A**) Distribution of cell types in the single-cell RNA sequencing data from N = 100,064 cells from the 26 breast cancer tumor samples, used to determine if a gene is expressed in immune cells. (**B**) Fraction of immune cell types in which the genes associated with the immunosuppressive CH mutations in TII cells, were expressed.

As an additional filter for potential passenger mutations, we exclude mutations in genes that are not expressed in immune cells since these will not affect immune function. We used scRNA-seq data from 100,064 cells from the breast cancer tumor microenvironment (Fig. 5A)^28^ to exclude mutations in genes not expressed in immune cells. The number of immune cells in which the genes in Table 1 are expressed, range from 603 for *KIF15* to 15,806 for *UBE2N* (Fig. 5B). These genes were expressed primarily in T-cells (55.3%) and myeloid cells (35.1%) (Fig. 5B).

We also excluded mutations in genes that are subject to hypermutation or extreme variability as part of the natural immune response or due to adaptations to different antigens. These include immunoglobulin, immunoglobulin-like receptor, histocompatibility antigen, and T-cell receptor genes.

Lastly, most secondary CH mutations (Fig. 1) are not likely to have a functional effect on the immune cell, even if protein-altering. For example, the effect of a single missense mutation or an in-frame insertion/deletion in an unstructured region of the protein will not always affect the structure or function of the protein. We used two mutation-significance prediction tools – sorting intolerant from tolerant (SIFT)^35^ and PolyPhen2^36^ – to select mutations that were predicted to be “deleterious” or “damaging” by both tools. SIFT predictions are primarily based on the degree of protein sequence conservation across homologous proteins. In addition to sequence conservation, PolyPhen2 incorporates the potential effect of the mutation on protein structure. Truncating and frame-shift mutations were also considered to be deleterious (SI Table S3). The pathogenicity prediction of SIFT and PolyPhen2 were consistent with predictions by CADD, a tool that integrates the annotations by multiple other predictors (SI Table S3)^37^. Gene pathway enrichment analysis using the Reactome pathway analysis tool^38^ identified 32 significantly over-represented pathways (false discovery rate (FDR) < 0.05). Of the 32 pathways, 17 were part of the Disease top-level pathway (SI Table S4).

Together, the above considerations maximize the likelihood that the selected mutations are pathogenic and not passenger mutations. Of the 47,255 CH mutations in TII, 1,710 (3.6%) frequently occurred in BRCA samples (> 5% of samples). Of these 1,710 mutations, 567 (33.2%) were rare mutations with gnomeAD allele frequency < 0.01%. Of these 567 mutations, 384 (67.7%) did not occur in genes that are naturally hypermutated or highly variable. Finally, of these 384 mutations, 95 (24.7%) were predicted to be deleterious or damaging by both SIFT and PolyPhen2 (SI Table S3) The cancer subtype, stage, and age distribution of these mutations (SI Figure S2) is similar to the overall distribution for all samples (SI Figure S1).

### Potential immunosuppressive mutations

Although the pathogenic variants identified above may affect cellular function, they may not necessarily affect anti-tumor immune response. To identify mutations that are likely to affect anti-tumor immune response, we considered two factors. First, we conducted a literature review to determine if there is in vivo or in vitro experimental evidence showing that the gene associated with each variant is involved in immune response. Second, we analyzed differentially expressed genes to confirm that immune system pathways could be affected by the variant. Eight potentially immunosuppressive CH mutations in TII cells were identified (Table 1). Studies suggesting an immunosuppressive effect of mutations in these genes are summarized below.

Core 1 Beta1,3-Galactosyltransferase 2 (*C1GALT1C1*, *COSMC*) encodes a chaperone protein required for the proper folding of T-synthase. T-synthase is required for the complete glycosylation of membrane glycoprotein Core 1 O-Glycan (T antigen)^39^. Incomplete glycosylation of T antigen results in the Tn antigen which has been associated with Tn syndrome, an autoimmune disease, and an immune suppressive microenvironment in colorectal cancer^40^. An analysis of blood cells from two patients with Tn syndrome and 25 healthy donors showed that the Tn syndrome was likely a result of somatic variants in *COSMC* which caused it to lose its chaperone function resulting in Tn antigens^41^. An in vitro study using the Jurkat cell line (T-lymphoblast) also showed that variants in *COSMC* results in the Tn positive phenotype^42^.

The dipeptidyl-peptidase IV (*DPP4*) is a protein found in the extracellular domain of CD26 that acts by cleaving N-terminal proline or alanine dipeptides at position two. CD26 is expressed as either a membrane-bound form that is typically expressed on CD4 + helper/memory T cells, or as a soluble form (sCD26) found in serum^43^. CD26 was also associated and co-expressed with adenosine deaminase (ADA) in Jurkat T cell lines, verified with in vitro binding assays demonstrating its binding via the extracellular domain of CD26. ADA deficiency causes severe combined immunodeficiency disease (SCID) in humans and demonstrates one of the immunopathological roles of *DPP4*^44^. *DPP4* also plays a role in fibrosis and immunoregulation and has received increasing attention in autoimmune diseases such as systemic lupus erythematosus (SLE) in which clinical evidence showed increased CD26 mRNA in SLE patients by 3.6-fold compared to controls^45^.

Eukaryotic Translation Initiation Factor 4E Binding Protein 1 (*EIF4EBP1, 4E-BP1*) encodes a translation repressor protein that directly interacts with eukaryotic translation initiation factor 4E (eIF4E). eIF4E is a component of a complex that recruits 40S ribosomal subunits to the 5’ end of mRNAs specific to monocytes. Dephosphorylation of *EIF4EBP1* leads to its interaction with eIF4E resulting in the inhibition of cap-dependent translation both in vivo and in vitro, playing a key role in human cancer^46^. *EIF4EBP1* phosphorylation was shown to regulate protein synthesis required for T-cell proliferation^47^. Immunohistochemical analysis showed increase expression of *EIF4EBP1* in subtypes of B-cell lymphoma and reactive lymphoid tissue^48^. Additionally, *EIF4EBP1* has been suggested to have a positive regulatory effect on autophagy through its regulation of mammalian target of rapamycin complex 1 (mTORC1). A study transfected a miR-99a-3p antagomir leading to negative regulation of autophagy. Thus, it not only plays a role in cancer, but due to its relationship with miR-99a-3p has a role in autoimmune diseases such as in SLE^49^.

Interestingly, four separate mutations in *EIF4EBP1* were included in our list of eight potentially immunosuppressive mutations. In addition, there was considerable overlap in the occurrence of *EIF4EBP1* mutations with 134 of 173 samples (77.46%) containing two or more of the four mutations. Since all these variants were detected in both tumor and matched blood samples with log odds (LOD) accuracy > 4.0, they are unlikely to be artifacts. For example, a sample with both the R63W and S65L variants had LODs of 44.02 and 54.99 for the two variants. Instead, it was more likely that the frequent occurrence of these mutations is because loci for two of the variants (R56W and R63W) contain CpG dinucleotides. Mutations in CpG dinucleotides are an order of magnitude more frequent than at other sites^50^. In addition, these four mutations are in the eIF4E binding site (residues 51–67)^51^. Phosphorylation of the protein in response to hormone signaling initiates mRNA translation. These mutations could disrupt mRNA translation, potentially affecting anti-tumor immune activity of the associated cells. Therefore, we speculate that the occurrence of these mutations may be correlated with the occurrence of cancer.

The protein expressed by the Kinesin Family Member 15 (*KIF15, NY-BR-62*) gene is part of a family of proteins that transport various cellular components such as organelles, protein complexes, and mRNA along microtubules. This protein has been implicated in the progression of various cancer types, including breast cancer^52^. One study found that *KIF15* was primarily expressed in inflammatory monocytes in the tumor microenvironment and was a prognostic marker for hepatocellular carcinoma^53^. Expression of *KIF15* was also found to increase B-cell proliferation in Burkitt lymphoma^54^.

Ubiquitination requires a ubiquitin activating E1 enzyme, an E2 ubiquitin conjugase, and an E3 ubiquitin ligase^55^. *UBE2N* is a K63-Ub-specific E2 enzyme that has been investigated for its role as a growth promoter of several human cancers such as breast cancer and neuroblastoma^56,57^. Gene expression analysis in human acute myeloid leukemia, implicated *UBE2N* as necessary for maintaining oncogenic immune signaling states. Suppression of *UBE2N* decreased oncogenic immune signaling, promoting cell death of leukemic hematopoietic stem and progenitor cells (HSPC) and ensured normal hematopoiesis^58^. *UBE2N* was also found to be essential for *RIG-I* mediated immune signaling in response to viral infection^59^.

Based on in vitro or in vivo experimental evidence described above, eight (5.3%) of the 95 potential pathogenic CH mutations in TII cells were identified as potentially affecting immune response (Table 1). As further support for a potential immune system related effect, RNA sequencing data for the BRCA samples were used to identify differentially expressed genes between samples with and without each mutation. For the significantly differentially expressed (SDE) genes (false discovery rate < 0.05), we then identified the associated top-level pathways in the Reactome pathways database^38^. A number of the SDE genes for each of the mutations were associated with immune system pathways, providing further support for an immunosuppressive role for these mutations (Table 2).

**Table 2:** Pathways affected by the potential immunosuppressive mutations. For each of these mutations, we identified significantly differentially expressed (SDE) genes (False Discovery Rate < 0.05). For each of these SDE genes we then identified the associated top-level Reactome pathway. The following table shows the number of SDE genes associated with each pathway. A number of SDE genes for each of the mutations are associated with the Immune System pathway, supporting an immunosuppressive role for these mutations.

Reactome top-level pathways	C1GALT1C1 V276G	DPP4 V354G	EIF4EBP2 D55H	EIF4EBP2 R56W	EIF4EBP2 R63W	EIF4EBP2 S65L	KIF15 T334P	UBE2N P63A
*Immune system*	*52*	*158*	*19*	*28*	*7*	*35*	*72*	*26*
Signal transduction	61	131	54	64	14	46	142	25
Metabolism	53	93	35	43	13	35	126	24
Gene expression	27	74	48	49	13	59	42	15
Metabolism of proteins	21	51	20	21	10	40	54	10
Developmental Biology	19	39	15	18	6	18	47	15
Disease	28	53	11	20	3	16	40	9
Vesicle-mediated transport	19	29	5	12	2	14	28	5
Cell cycle	6	17	7	11	2	43	7	2
Homeostasis	19	36	9	13	1	8	36	12
Cellular response to stress	8	23	7	6	1	13	14	3
Organelle biogenesys and maintenance	7	18	2	4	2	11	13	6
Neuronal systems	13	13	6	8	2	3	19	2
DNA repair	11	18	3	4	0	12	10	0
Extracellular matrix organization	7	10	1	3	0	0	27	4
Chromatin organization	4	11	6	11	0	4	7	2
Muscle contraction	6	3	2	6	2	1	18	2
Programmed cell death	4	8	0	1	2	8	6	2
Cell–cell communication	4	8	3	3	0	0	9	2
DNA replication	1	4	0	0	1	12	0	0
Circadian clock	4	3	0	0	1	1	1	1
Reproduction	0	1	1	2	0	0	3	1
Mitophagy	0	1	0	0	1	1	0	0

## Discussion

*Tumor cell* mutations that inhibit anti-tumor immune response have been extensively studied^5–7^. However, mutations in *immune cells* that could affect anti-tumor immune response, to our knowledge, have not been previously studied. There are two reasons for this. One, cancer genomics research has been primarily focused on finding mutations in tumor cells that “drive” tumor growth^34^. Two, under normal hematopoiesis immune cells are unlikely to accumulate somatic mutations in a significant fraction of immune cells due to constant replacement from hematopoietic stem and progenitor cells^8^. However, a relatively recently discovered mechanism, clonal hematopoiesis, does in fact result in the accumulation of somatic mutations in a significant proportion (> 2%) of immune cells^9,10^. The incidence of clonal hematopoiesis increases with age (> 10% of population over 70 years)^16^, occurs frequently in TII cells (14–65% of study samples)^17–21^, and has been implicated in hematologic^14^ and cardiovascular^15^ malignancies. However, clonal hematopoiesis does not by itself result in these malignancies. Secondary mutations are required to produce a disease state^14^, as illustrated in Fig. 1. For example, mutations in genes associated with Clonal Hematopoiesis of Indeterminate Potential (CHIP) (*DNMT3A, TET2*, etc.) by themselves are not immunosuppressive. Additional secondary mutations are required for pathogenicity^9^. Mutations in the top 10 CHIP genes were not included in our set of potentially immunosuppressive mutations because they did not occur in at least 5% of the breast cancer cases, one of our selection criteria (SI Table S1). There may be as many as 70 genes that “drive” clonal expansion^22^**.**

Although CHIP mutations cannot “drive” tumor growth, it is possible that secondary mutations in clonally expanding immune cells could affect their anti-tumor activity, potentially “facilitating” or indirectly increasing the risk of tumor growth. Secondary immunosuppressive mutations in a significant number (2–25%) of TII cells due to clonal hematopoiesis would represent some degree of immunodeficiency, which could affect the progression of solid cancers. There were many instances where the VAF in the tumor (or matched blood) sample was significantly higher than in the matched blood (or tumor) sample (Fig. 4A). We offer two possible explanations. The tumor microenvironment was enriched (or limited) in the specific immune cell subtype containing the clonal hematopoietic mutation, due to the nature of the immune response. Alternatively, the tumor microenvironment varies significantly between regions of the tumor^60^, with vastly different immune infiltration in different regions. In stage 3 of our approach, we identified ninety-five *potential* pathogenic non-passenger CH mutations in TII cells. Further investigation may reveal that a combination of these mutations could represent a polygenic risk marker for breast cancer.

In stage 4 of our approach, we identified a set of eight *potential* immunosuppressive clonal hematopoietic mutations in tumor infiltrating immune cells (Table 1). Highly restrictive criteria were used to select these potential immunosuppressive mutations. It is likely that these restrictive criteria resulted in the exclusion of other potential immunosuppressive mutations. However, as a first step it was important to identify the most likely candidates for subsequent in vitro and in vivo experiments required to confirm their effect on immune response. Without additional experimental validation the immunosuppressive role of these mutations remains speculative. It is possible that the mutations identified here are passenger mutations that do not affect gene function in the context of tumor growth, despite the multiple highly restrictive criteria designed to prevent the selection of passenger mutations. However, if an immunosuppressive effect of these mutations in TII cells is experimentally confirmed it could represent a novel paradigm in our understanding of cancer progression.

## Conclusions

This study is a first step towards investigating the potential role of clonal hematopoietic (CH) mutations in tumor infiltrating immune (TII) cells, on anti-tumor immune response. Mutations in immune cells, unlike mutations in tumor cells, cannot drive tumor growth. However, it is possible that mutations, in a significant proportion (2–25%) of TII cells, that inhibit an effective immune response could facilitate tumor growth. Out of over 4 million different mutations in 1,064 breast invasive carcinoma matched tumor and blood samples, we have identified a set of eight mutations that were clonally expanded, tumor infiltrating, and potentially immunosuppressive. Multiple highly restrictive criteria were used to exclude potential passenger mutations while selecting for potential immunosuppressive mutations. Further in vitro and in vivo investigations are needed to confirm that these mutations inhibit anti-tumor immune response. With experimental validation, the role of immunosuppressive CH mutations in TII cells could represent a novel paradigm in the etiology of cancer.

## Methods

Exome sequencing data for 1,064 breast invasive carcinoma (BRCA) samples from the cancer genome atlas (TCGA)^27^ were used to identify mutations in tumor infiltrating immune (TII) cells. The sequencing data includes data from matched tumor and normal blood samples. The “protected” or “controlled access” mutation accumulation format (MAF) file^61^ containing mutations called using the genome analysis toolkit (GATK) version 2.4 variant caller^62^, was downloaded from TCGA. These protected files include mutations that are filtered out in publicly accessible databases, such as cBioPortal, to prevent donor identification. The protected files include mutations in normal blood sample which are considered potential germline mutations that could identify specific individuals. However, for this study we obtained permission to use the protected data so that we could investigate somatic mutations in immune cells. The MAF file includes read counts for all alleles, which were used to calculate variant allele fractions (VAF). Only somatic variants were considered in this study, and all variants were supported by reads in both the tumor sample and matched blood sample. For this analysis we selected variants with a minimum Log Odds (LOD) ratio of 4.0, corresponding to a 10^4^:1 odds of correctly detecting variants, and a minimum base quality score of 10^63^. The LOD for selected variants ranged from 4.01–75.10 and sequencing depth ranged from 16–725. The filtering criteria used to limit the possibility of sequencing artifacts are discussed further in the SI Text.

Gene expression data from RNA sequencing data from the BRCA samples were downloaded from TCGA to identify differentially expressed genes. Clinical data were also downloaded from TCGA to assess demographic and tumor characteristics of the data used.

Single-cell RNA sequencing (scRNA-seq) data were used to confirm that the genes containing mutations in TII cells are expressed in immune cells. scRNA-seq data for 26 breast cancer cases were downloaded from the genome exchange omnibus (GEO), GSE176078^28^. Cell-type and barcoded single cell expression data from these datasets were used to determine the cell-types in which a given gene is expressed.

The genome aggregation database (gnomAD v2.1) contains variant information from over 100,000 genomes^64^ The allele population frequency in gnomAD was used to identify potentially pathogenic mutations. For this analysis, mutations with allele population frequency of less than 0.0001 were considered to be potentially pathogenic.

Two mutation significance prediction tools – SIFT^35^ and PolyPhen2^36^ – were used to determine if a given mutation is likely to affect protein function. SIFT predictions are primarily based on protein sequence conservation and the differences in characteristics of the wild type and mutant amino acid. PolyPhen2 combines multiple sequence and structural features to predict the potential effect of missense mutations. Structural features are determined by homology modeling.

The python statsmodels v0.13.2 was used to perform all statistical analysis. *p* value calculations for significantly differentially expressed genes used the two-sided Welch *t* test with Satterthwait degrees of freedom for independent samples with unequal standard deviations. False discovery rate calculations used the Benjamini–Hochberg correction for multiple testing with independent samples. 95% confidence interval for VAF was calculated using the binomial proportion function.

### Supplementary Information

Supplementary Information 1.

Supplementary Information 2.

## Data Availability

The datasets analyzed for this study can be found in the cancer genome atlas (TCGA) (https://portal.gdc.cancer.gov/repository, dbGaP accession number phs000178) and the gene exchange omnibus (https://www.ncbi.nlm.nih.gov/geo/, accession number GSE176078) repositories.
