# Supplementary material for: Potential immunosuppressive clonal hematopoietic mutations in tumor infiltrating immune cells in breast invasive carcinoma
Source: Sci Rep. 2023 Aug 12;13:13131. doi: 10.1038/s41598-023-40256-9 (PMC10423211; doi:10.1038/s41598-023-40256-9)
Supplement: Supplementary file 1 — Supplementary Information 1. [file 41598_2023_40256_MOESM1_ESM.docx]

Potential immunosuppressive clonal hematopoietic mutations in tumor infiltrating immune cells in breast invasive carcinoma

**Supplementary Information**

This document contains additional information referenced in the main text.

**1. Filtering criteria for variant calling.**

To limit the possibility of sequencing artifacts, we select variants with called log odds (LOD) accuracy > 4.0. In addition, we selected variants that were detected in both tumor and matched blood samples from the same subject. A recent study in Blood ^1^ discusses the importance of filtering out potential sequencing artifacts when trying to detect clonal hematopoietic mutations. Although the Blood study examines CHIP mutations called from a single sample per subject, it is instructive to compare our filtering criteria with matched samples to that used by the single-sample Blood study. Following is a comparison to each of the filtering criteria used in the Blood study:

- Total read depth >= 20. Our total read depth across both samples per subject was >= 37.
- Alternate allele read depth > 2. Our allele read depth across both samples per subject was > 2.
- Variants supported by forward and reverse reads. Mutect2, the variant caller used here, filters out variants not supported by forward and reverse reads, while allowing for strand bias near the ends of exome targets.
- Variant allele fraction (VAF) > 2%. We also selected variants with VAF > 2%.
- Correlation with age or the TERT promoter variant rs7705526 to identify clonal hematopoietic mutations of indeterminate potential (CHIP) variants. We did not consider these criteria since we are interested in variants correlated with cancer which may not be CHIP variants.
- VAF < 50% to exclude germline variants. We used a more conservative VAF < 25% to exclude germline variants.
- Variant quality checks. We excluded variants with base quality < 10.
- Sample quality based on number of mutations. We did not consider this criterion since we are working with tumor samples and a large number of mutations are to be expected.

**2. Clinical and variant profile of breast cancer samples.**

The proportion of samples by breast cancer subtype, age, cancer stage and variant type are shown in Fig. S1.

**3. Clinical profile of potential immunosuppressive mutations.**


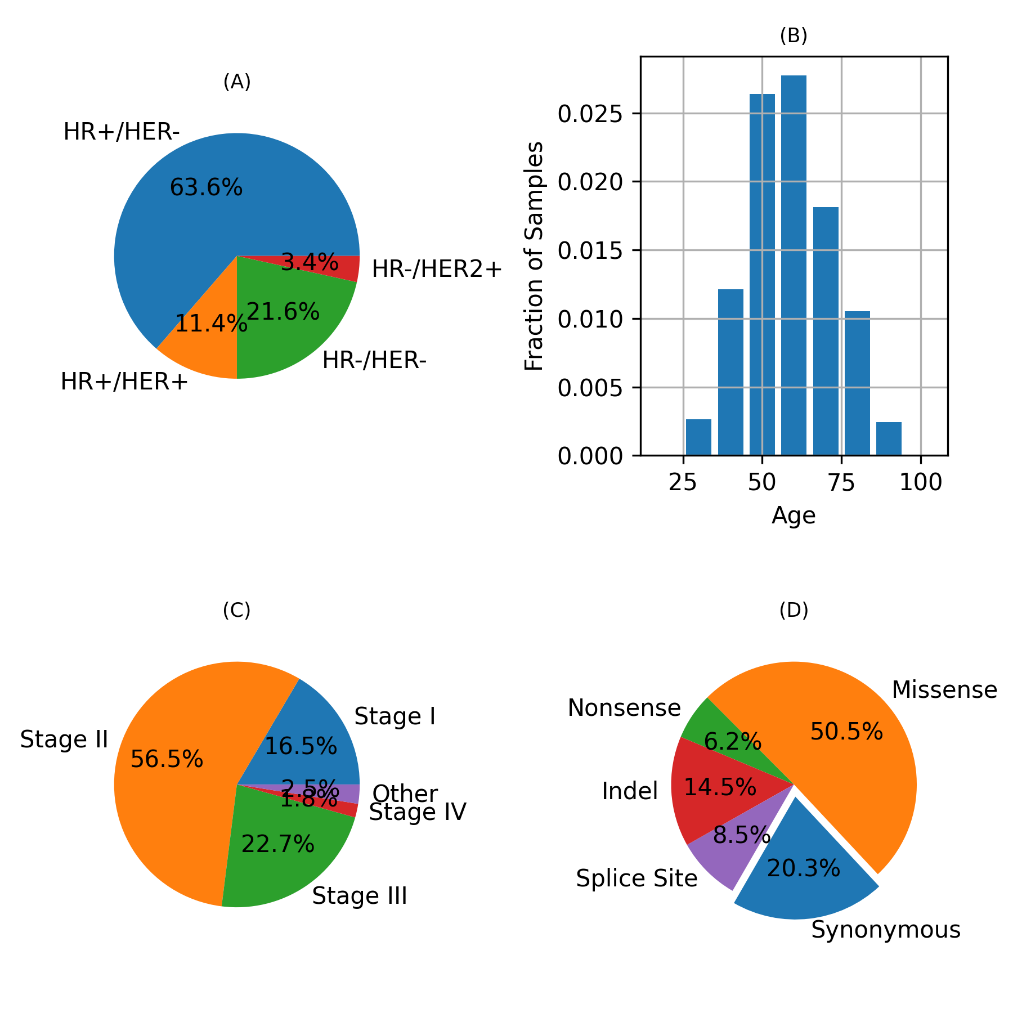


*Figure S1. TCGA BRCA clinical profile. (A) Breast cancer subtypes. (B) Age distribution. (C) Cancer stage. (A-C) N=1,064 samples. (D) Protein altering (non-synonymous and splice site) mutations selected in Stage 1 of our approach. N=558,470 mutations.*

The proportion of samples by breast cancer subtype, age, and cancer stage for samples containing the potentially immunosuppressive CH mutations in TII cells are shown in Fig. S2.


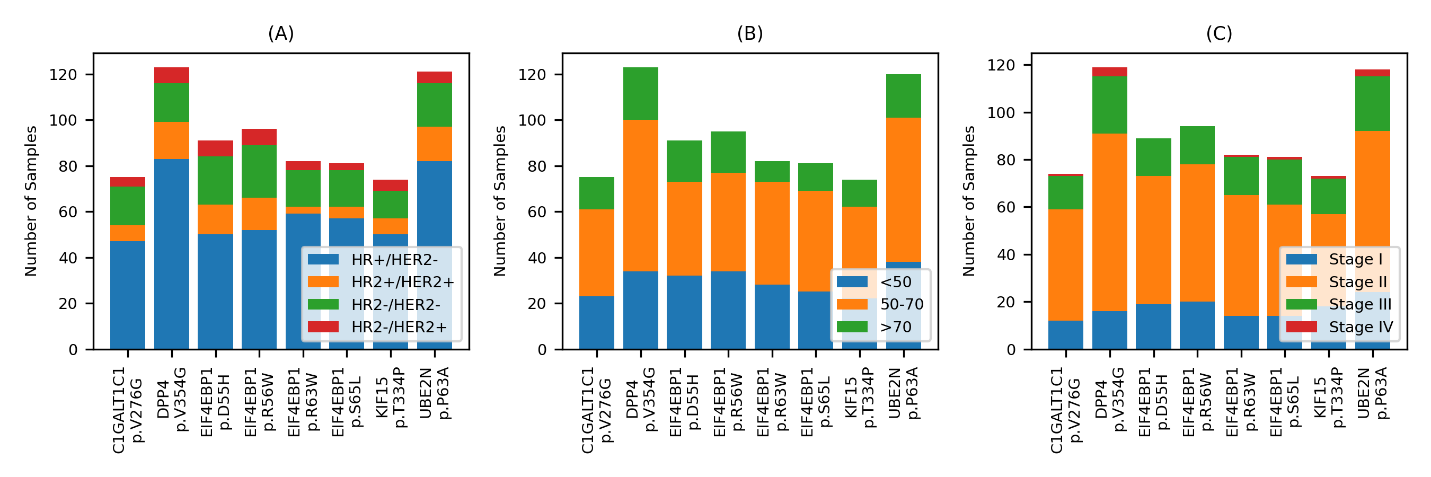


*Figure S2. Clinical profile of immunosuppressive mutations. Distribution of samples by subtype (A), age group (B), and cancer stage (C).*

**References**

1. Vlasschaert C, Mack T, Heimlich JB, et al. A practical approach to curate clonal hematopoiesis of indeterminate potential in human genetic data sets. *Blood*. 2023;141(18):2214-2223. doi:10.1182/BLOOD.2022018825
